# Musculoskeletal disorders and their associations with health- and work-related factors: a cross-sectional comparison between Swedish air force personnel and army soldiers

**DOI:** 10.1186/s12891-020-03251-z

**Published:** 2020-05-14

**Authors:** Matthias Tegern, Ulrika Aasa, Björn O. Äng, Helena Larsson

**Affiliations:** 1 Department of Community Medicine and Rehabilitation, Unit of Physiotherapy, Umeå University, Umeå, Sweden; 2 Department of Neurobiology, Care Sciences and Society, Division of Physiotherapy, Karolinska Institutet, Huddinge, Sweden; 3 School of Education, Health and Social Studies, Dalarna University, Falun, Sweden; 4 Centre for Clinical Research Dalarna – Uppsala University, Falun, Sweden; 5 Swedish Armed Forces, HQ, Stockholm, Sweden

**Keywords:** Musculoskeletal pain, Musculoskeletal injuries, Fighter pilots, Helicopter pilots, Rear crew, Deployment, Army

## Abstract

**Background:**

The high numbers of musculoskeletal disorders (MSD) among soldiers in the Swedish Armed Forces has led to the implementation of an effective prevention program, the musculoskeletal screening protocol (MSP), including questionnaires, physical tests and individual intervention of their MSD. A corresponding MSP for the Swedish Air Force is also needed due to earlier reported high prevalence of MSD. We therefore investigated the prevalence of MSD in Swedish Air Force personnel (AF) and compared this to Swedish Army deployed soldiers (DS). Individual, health- and work-related factors associated with MSD were also investigated.

**Methods:**

Cross-sectional questionnaire-based study on 166 male AF and 185 DS. AF consisted of fighter pilots, helicopter pilots and rear crew from one Swedish air base.

**Results:**

The one-year and point prevalence, respectively, of MSD were significantly higher for AF compared to DS with regard to both the upper quarter of the body (i.e. neck, shoulder and thoracic regions) (AF = 54.8 and 31.3%, DS = 26.1 and 13.6%, *p* = 0.01) and the lumbar region (AF = 38.0 and 18.7%, DS = 22.2 and 7.1%, *p* = 0.00). No significant differences were present between fighter pilots, helicopter pilots and rear crew regarding MSD prevalence.

Factors significantly associated with having both upper quarter and lumbar regions MSD were group (i.e. greater odds for AF than DS) and self-reported physical health as less than excellent. Additionally, being older and taller were also factors associated with lumbar region MSD.

**Discussion:**

Despite a generally healthy lifestyle, MSD were commonly reported by AF and DS, with generally higher prevalence in AF who mainly reported MSD in the upper quarter of the body. The results from this study indicate that the MSP can be a meaningful tool to prevent MSD in air force personnel and that questions regarding general health and MSD in specific body regions should be included in screening protocols. The development of the preventive program MSP is therefore recommended for the Swedish Air Force.

## Background

Musculoskeletal disorders (MSD) are a widespread problem in armed forces worldwide [1], especially in air force personnel [2–4] and army soldiers [5–8]. Recently it was reported that MSD have increased over time among Swedish deployed soldiers (DS) [9] and Dutch Air Force personnel [10]. This trend is worrisome given that MSD may negatively affect performance of military personnel [11]. Further, the presence of pain may affect motor control [12] and can be seen as a safety issue [13]. Prevention of MSD is therefore crucial to maintaining a high readiness of armed forces and strategies to reduce the high rates of MSD in the Swedish Armed Forces have been developed for army soldiers.

One such preventive strategy is the implementation of the Musculoskeletal Screening Protocol (MSP) in Swedish Armed Forces for army soldiers. The MSP involves screening soldiers for established risk factors and early signs of MSD [14]. This includes a questionnaire and physical tests mainly focusing on the lower extremities and general physical performance tests [15, 16]. Together, the questionnaire and the tests identify soldiers needing early rehabilitation and/or an individual physical training program to improve their functional ability. The MSP evaluates musculoskeletal pain or problems in 10 anatomical regions, lifestyle factors and functional limitations. The tests of function include measures of passive muscle flexibility as well as muscular strength and endurance. Soldiers who report pain are referred to medical care and soldiers who are not sufficiently strong or flexible, or do not show optimal sensorimotor control of the knees, receive individually-tailored intervention programs based on their screening outcomes. In a study including 862 Swedish Armed Forces soldiers, the implementation of MSP reduced the discharge rate from basic military training by approximately 50% [14]. Therefore, all Swedish soldiers now undergo screening when entering basic military training and prior to deployment to international missions according to the MSP since its implementation in 2010. A similar system for the Swedish Air Force (AF) personnel has not been implemented but is under development. In order to develop the MSP for the Swedish AF, based on the successful protocol for army soldiers, the extent and burden of MSD among AF personnel needs to be established and related to the extent among army soldiers. Studies comparing the prevalence of MSD between the Swedish AF and other military occupations are, however, lacking.

To develop the MSP so that it can also capture risk factors for the development of MSD and enable individualized treatment also for personnel in the AF, similarities and differences between personnel in the AF and the Army need to be examined. So far, research has shown increased odds for neck pain among pilots compared to army officers in NATO countries [17]. Also in military pilots, exposure to various work-related and individual factors has been suggested as increasing the risk for developing MSD [18]. For neck pain, these include high accelerations [19], helmet-mounted equipment [20–22], disadvantageous postures or work tasks [23] and head movements in the cockpit [24]. Factors associated with low back pain include ergonomics of the cockpit and work posture [10, 25] and individual factors such as back muscle function [26] and age [3]. Associated factors related to the high rates of low back pain among army soldiers [5–7, 27] include previous injuries, anthropometrics and level of fitness training [6, 28]. Further, regarding work-related psychosocial factors, specifically a low self-reported mental health or being mentally unprepared, has been associated with premature discharge from military service in Sweden [14, 16]. Also, lack of support from leaders and mental stress have been associated with low-back pain among Danish deployed soldiers [27], thus supporting the multifactorial origins of MSD.

The present study aimed to investigate the prevalence of MSD in Swedish AF and army soldiers. Further, we aimed to investigate associated individual, health- and work-related factors with MSD.

## Methods

### Study design

This was a cross-sectional questionnaire-based study investigating prevalence of MSD in Swedish air force personnel (i.e. AF) and Swedish Army deployed soldiers (hereafter abbreviated DS). Individual, health-and work-related factors associated with MSD in the upper body (i.e. the neck, the thoracic region and the shoulders) and lumbar region were studied. The AF answered the questionnaires when the researchers visited the respective air base, while the DS answered the questionnaire when they were undergoing medical checks in preparation for international deployment to Afghanistan in 2012 [9].

Both oral and written information were given in accordance to the Helsinki declaration, and all included AF and DS gave their written informed consent. The oral information was provided during a briefing by one of the authors (HL) who described the procedure of the MSP and informed all individuals that they would receive any needed follow-up based on their screening outcomes even if they declined to participate in the research. The regional ethics committee in Stockholm approved the study, DNR:2010/1423–31/5, DNR:2011/928–32, DNR:2013/144–31/2.

### Participants

**Fig. 1:**
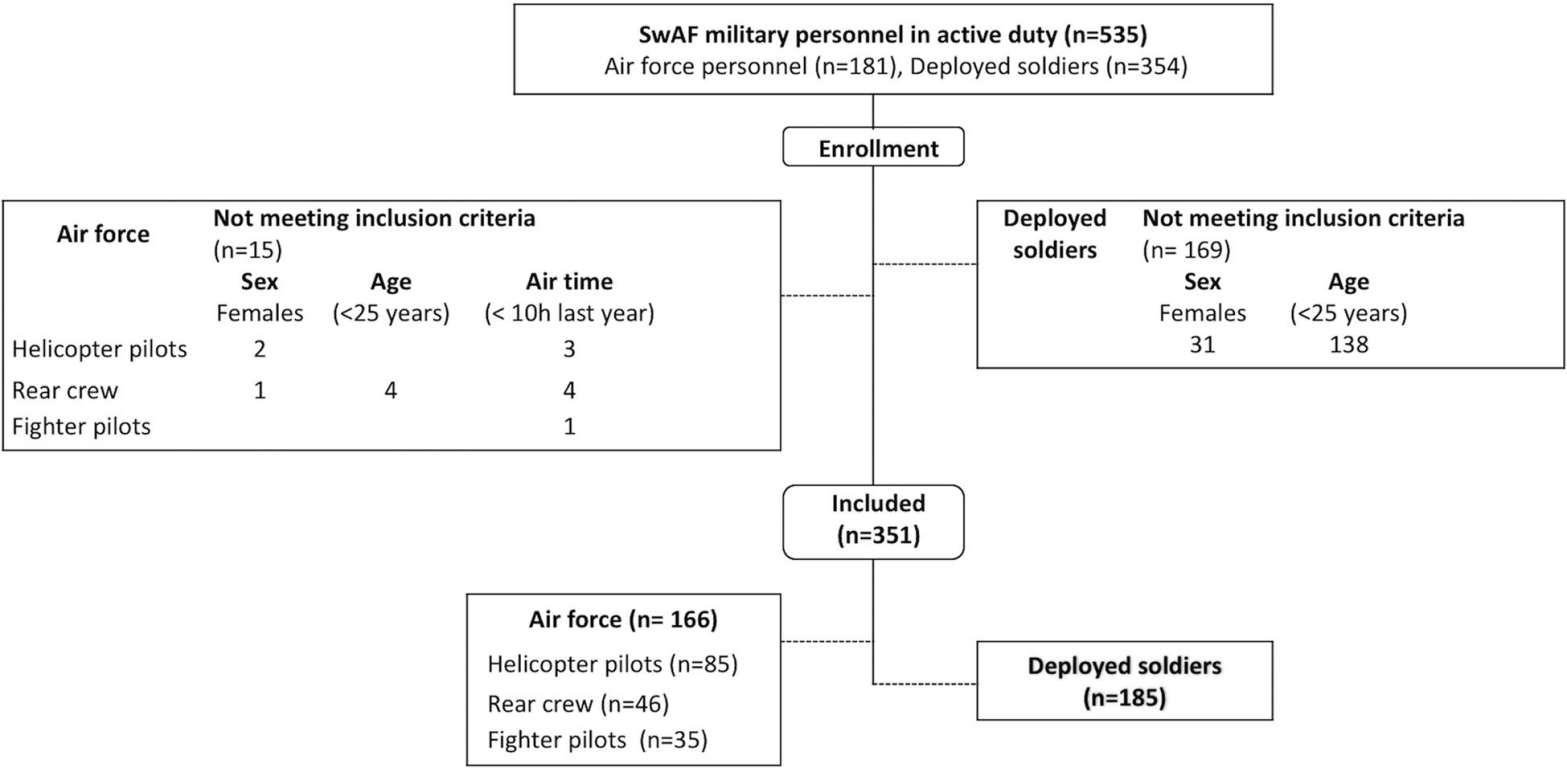
Description of inclusion of participants

All AF and DS personnel in active duty (*n* = 535) were asked to participate in the study. The AF cohort were recruited from two Swedish air bases and included fighter pilots (FP), helicopter pilots (HP) and rear crew (RC). From the first air base, all HP and RC in duty were included during 2013. From the second air base, all HP and RC together with FP were included during 2016. The DS were employed as army soldiers or officers at one army unit and were undergoing medical checks in preparation for deployment. The AF (*n* = 181, mean (SD) age 38 [9] years), as a cohort, were significantly (*p* = 0.01) older than the DS (*n* = 354, mean (SD) age 29 [9] and few females (n = 3) were in service at the two air bases. Therefore, only males aged 25 years and older were included in the analyses (AF *n* = 166, DS *n* = 185, total *n* = 351), Fig. 1.

### Measurement

The questionnaire in this study is also used as part of the MSP among Swedish armed forces personnel to capture early signs of, as well as to prevent the incidence of, MSD. The questionnaire has previously been described in detail [16] and was somewhat modified for this study to include additional flight-related questions.

### Anthropometrics

The participants reported age (years), body height (m) and body weight (kg). Body mass index (BMI) was calculated as kg/m^2^.

### Musculoskeletal disorders

Previous studies using the MSP have used the term musculoskeletal complaints or injuries (MSCI) [14–16]. In this study, this is synonymous with MSD. To establish the one-year and point prevalence of MSD, respectively, the participants answered the question:

“Have you during the past 12 months had occasional or more persistent complaints from, or an injury to, any part of your body? Do you still have these at present?” with answers yes or no. Ten predefined body regions were used: the neck, the thoracic region (i.e. between the scapuale, the lumbar region, the shoulders, the elbows, the hands, the hips/pelvis, the knees, the lower leg and the feet. We combined the neck, the thoracic region and the shoulders into “upper quarter of the body” (also denoted “upper quarter”) in our analyses due to the multiple anatomical structures combining them. The hips/pelvis, knees, lower leg and feet were combined into “lower extremity”. The total frequency for anywhere in the body was reported as “any region”. Further, participants rated the current intensity of pain regarding their MSD using an 11-point numerical pain rating scale (NPRS). Any sick-listing or if they had taken time off work due to their MSD was answered with yes or no.

### Health- and work-related questions

To assess their self-rated general health, the following questions were used: How do you experience your [1] “physical health” [2] “mental health” [3] “social environment” [4] “physical environment” and [5] “work ability”? A seven-point scale with answers ranging from “very poor” to “excellent, cannot be better” was used. In the analyses, the answers were collapsed and coded into: poor (≤3), good [4, 5], or excellent (≥6) according to Larsson et al. [16]. As described by Monnier et al. [29], a “less-than-optimal” work ability was considered a risk in these environments. We dichotomized the answers from all five questions for the logistic analyses into: less than excellent and excellent. Tobacco use was assessed by the questions: “Do you use smokeless tobacco (i.e. chewing tobacco/ Swedish snus)?” and “Do you smoke?” with answers yes or no.

Motivation and preparation to perform their work tasks were assessed by the questions: “Are you motivated to perform your work tasks?”, “Are you sufficiently mentally prepared to perform your work tasks?”, “Are you sufficiently physically prepared to perform your work tasks?”, with answers yes or no. The AF also reported their accumulated air-time and annual air-time, respectively.

### Physical activity level

Physical activity level during leisure time was measured with two questions according to weekly occurrence on two intensity levels [1]; high/average, or [2] low intensity. A 5-point self-report scale was used with answers; never, irregular, 1, 2 or ≥ 3 times per week. In accordance to Larsson et al. [14, 16], this rating was added and converted to a score ranging from 0 to 16 points and thereafter grouped into; “low/inactive” (≤5), “active” [6–11], and “highly active” (≥12).

### Statistical analysis

Descriptive data were presented as numbers, percentages and means/medians with standard deviation (SD)/interquartile range (IQR) where appropriate. One-way Analysis of variance (ANOVA) and Student’s t-test were used to analyse continuous data, i.e. differences regarding age, height, weight and body mass index (BMI). The Kruskal Wallis test and Mann Whitney test were used for categorical variables, maximal pain intensity rating (NPRS) and total/annual air-time. The Chi-square test or Fisher’s exact test were used for dichotomous variables. Bonferroni corrections were applied when needed. Data were analysed and presented for each category of AF (i.e. FP, HP and RC, respectively) and all AF categories were then combined to form one category for comparisons to DS.

Logistic regression analyses were used to examine any relation between the independent variables from the questionnaire with the dependent variables of MSD during the last 12 months in the “upper quarter of the body” and MSD during the last 12 months in the “lumbar region”, respectively. The associations were reported as Odds Ratios (OR) with corresponding 95% Confidence Intervals (CI). Independent variables for multiple logistic regression were selected and forwarded through purposeful selection [30]. In a first step, univariate logistic regression was performed to identify independent variables associated (*p* < 0.20) with the dependent variable. These associated variables were then included in the multiple logistic regression model. A step-wise backward deletion process removing all non-significant (*p*-value > 0.05) variables was performed, leaving only variables significantly associated with MSD in the upper body or lumbar region, respectively. Body height was converted from meters to centimetres in the logistic regression to enhance the interpretation of odds ratios.

All calculations were performed using IBM SPSS Statistics for Windows, version 23 (IBM Corp., Armonk, N.Y., USA). A *p*-value < 0.05 was considered statistically significant.

## Results

### Prevalence of MSD in AF and DS

Table 1 shows the one-year and point prevalence of MSD for all body regions in AF and DS, respectively. A significantly higher one-year prevalence of MSD was reported for the AF compared to DS in the neck, thoracic shoulder and the combined upper quarter region (*p* < 0.01) as well as the lumbar region (*p* < 0.01). The numbers for the combined lower extremities region were slightly higher in DS, although not significantly different (*p* = 0.08). The point prevalence was significantly higher for the AF compared to DS in the neck (*p* = 0.01), shoulder (*p* = 0.03) and the combined upper body region (*p* < 0.01), as well as the lumbar region (p < 0.01) and any region (*p* = 0.01.

**Table 1:** Prevalence of musculoskeletal disorders in air force and deployed soldiers

		Air force		Deployed soldiers		
		*n* = 166		*n* = 185		
**One-year prevalence**	% (95% CI)					p-value
Neck		**27.7**	(21.5–35)	**10.8**	(7.1–16.1)	<.01
Thoracic		**31.3**	(24.8–38.7)	**12.4**	(8.4–18)	<.01
Lumbar		**38**	(30.9–45.5)	**22.2**	(16.8–28.7)	<.01
Shoulder		**24.1**	(18.2–31.1)	**12.5**	(8.5–18.1)	.01
Elbow		8.4	(5.1–13.7)	7.1	(4.2–11.7)	.69
Hand		7.8	(4.6–12.9)	5.4	(3–9.7)	.40
Hip		10.2	(6.5–15.8)	4.9	(2.6–9.1)	.07
Knee		21.1	(15.6–27.9)	22.2	(16.8–28.7)	.90
Lower leg		6.6	(3.7–11.5)	10.3	(6.7–15.5)	.26
Foot		11.5	(7.5–17.2)	16.8	(12.1–22.8)	.17
*Combined regions*						
Any region		80.7	(74.1–86)	71.9	(65–77.9)	.06
Upper quarter		**54.8**	(47.2–62.2)	**26.1**	(20.3–32.9)	<.01
Lower extremity		34.9	(28.1–42.5)	44.3	(37.3–51.5)	.08
**Point prevalence**						
Neck		**14.5**	(9.9–20.6)	**5.6**	(3.4–10.3)	.01
Thoracic		12.1	(7.9–17.9)	7.6	(4.6–12.3)	.20
Lumbar		**18.7**	(13.5–25.3)	**7.1**	(4.2–11.7)	<.01
Shoulder		**13.9**	(9.4–19.9)	**6.5**	(3.8–11.1)	.03
Elbow		4.8	(2.5–9.2)	3.3	(1.5–6.9)	.59
Hand		3.6	(1.7–7.7)	3.8	(1.9–7.6)	1.00
Hip		6.0	(3.3–10.7)	3.3	(1.5–7)	.31
Knee		13.9	(9.4–19.9)	10.3	(6.7–15.6)	.33
Lower leg		4.2	(2.1–8.5)	5.4	(3–9.7)	.63
Foot		8.4	(5.1–13.7)	8.7	(5.4–13.6)	1.00
*Combined regions*						
Any region		**53.6**	(46–61)	**40**	(33.2–47.2)	.01
Upper quarter		**31.3**	(24.8–38.7)	**13.6**	(9.4–19.3)	<.01
Lower extremity		25.3	(19.3–32.4)	23.5	(17.9–30.2)	.71

Numbers in bold indicate significant difference between groups (*p* < 0.05). Upper quarter: neck, shoulder and thoracic region. Lower extremity: hip, knee, lower leg, foot. *CI* Confidence Interval. A maximum of *n* = 2 missing data in one single body region

**Fig. 2:**
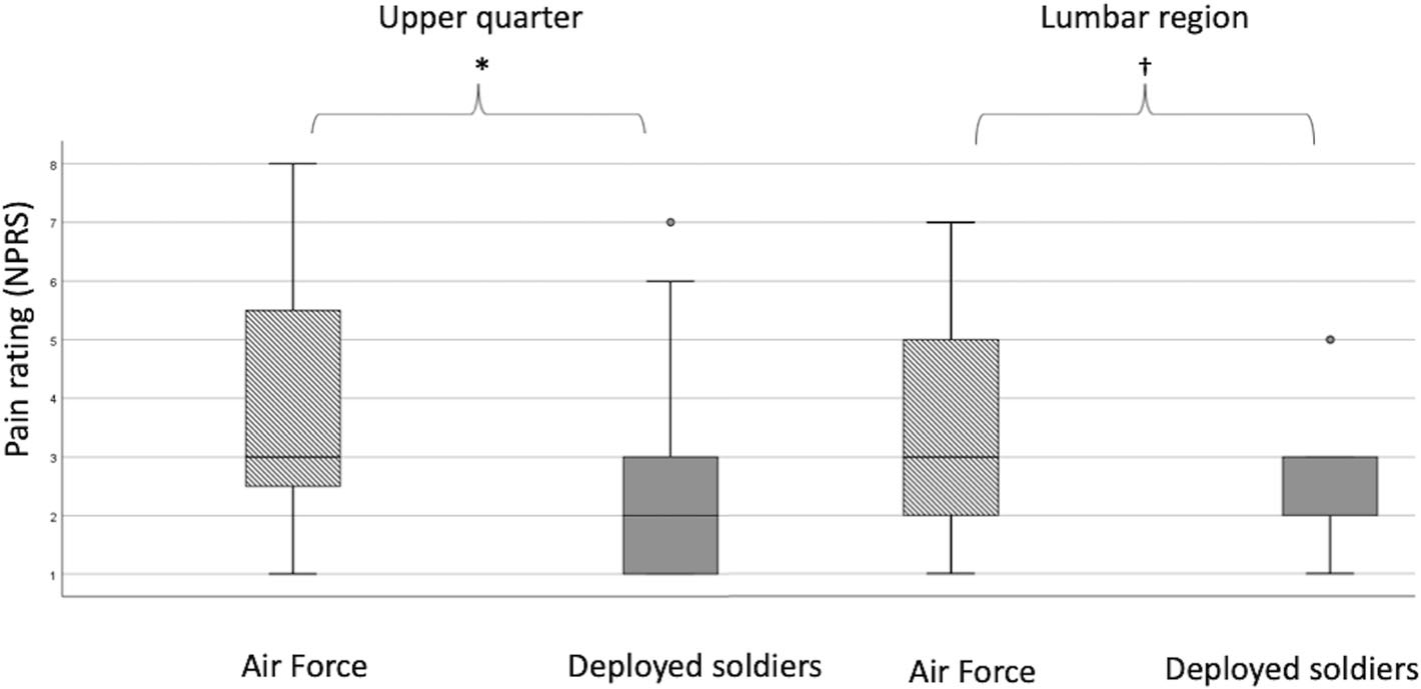
Box-plots showing the maximal pain rating using numerical pain rating scale for those who reported upper quarter (air force *n* = 52 deployed soldiers, *n* = 25) and lumbar region (air force *n* = 31, deployed soldiers, *n* = 13) MSD *, significant difference between AF and DS p < 0.01; ^†^, significant difference between AF and DS, p = 0.03

Figure 2 shows that the maximal pain intensity for both the upper body and lumbar region, respectively, was significantly higher for AF compared to DS (p < 0.01 and p = 0.03, respectively).

### Prevalence of MSD in FP, HP and RC

Table 2 shows the one-year and point prevalence of MSD for all body regions in air force personnel. No significant differences were found between groups.

**Table 2:** Prevalence of musculoskeletal disorders for fighter pilots, helicopter pilots and rear crew

		Fighter pilots		Helicopter pilots		Rear crew		
		*n* = 35		*n* = 85		*n* = 46		
**One-year prevalence**	% (95% CI)							p-value
Neck		37.1	(23.2–53.7)	22.4	(14.8–32.3)	30.4	(19.1–44.8)	.23
Thoracic		40	(25.6–56.4)	29.4	(20.8–39.8)	28.3	(17.3–42.6)	.47
Lumbar		42.9	(28–59.2)	31.8	(22.8–42.3)	45.7	(32.2–59.8)	.23
Shoulder		20.0	(10–35.9)	24.7	(16.8–34.8)	26.1	(15.6–40.3)	.84
Elbow		5.7	(1.6–18.6)	4.7	(1.9–11.5)	17.4	(9.1–30.7)	.06
Hand		11.4	(4.5–26.0)	5.9	(2.5–13.0)	8.7	(3.4–20.3)	.56
Hip		8.6	(3–22.4)	9.4	(4.9–17.5)	13.0	(6.1–25.7)	.79
Knee		17.1	(8.1–32.7)	25.9	(17.8–36.1)	15.2	(7.6–28.2)	.34
Lower leg		2.9	(0.5–14.5)	8.2	(4.1–16.0)	6.5	(2.2–17.5)	.65
Foot		11.4	(4.5–26.0)	14.1	(8.3–23.1)	6.5	(2.2–17.5)	.43
*Combined regions*								
Any region		88.6	(74.1–95.5)	76.5	(66.4–84.2)	82.6	(69.3–90.1)	.30
Upper quarter		60.0	(43.6–74.5)	51.8	(41.3–62.1)	56.5	(42.3–69.8)	.69
Lower extremity		28.6	(16.3–45.1)	42.4	(32.4–53.0)	26.1	(15.6–40.3)	.13
**Point prevalence**								
Neck		14.3	(6.3–29.4)	14.1	(8.3–23.1)	15.2	(7.6–28.2)	1.00
Thoracic		17.1	(8.1–32.7)	10.6	(5.7–18.9)	10.9	(4.7–23.0)	.60
Lumbar		22.9	(12.1–39)	12.9	(7.4–21.7)	26.1	(15.6–40.3)	.15
Shoulder		11.4	(4.5–26)	11.8	(6.5–20.3)	19.6	(10.7–33.2)	.44
Elbow		5.7	(1.6–18.6)	2.4	(0.7–8.2)	8.7	(3.4–20.3)	.24
Hand		2.9	(0.5–14.5)	3.5	(1.2–9.9)	4.4	(1.2–14.5)	1.00
Hip		5.7	(1.6–18.6)	5.9	(2.5–13)	6.5	(2.2–17.5)	1.00
Knee		14.3	(6.3–29.4)	17.7	(11–27.1)	6.5	(2.2–17.5)	.09
Lower leg		2.9	(0.5–14.5)	5.9	(2.5–13)	2.2	(0.4–11.3)	.69
Foot		11.4	(4.5–26)	10.6	(5.7–18.9)	2.2	(0.4–11.3)	.19
*Combined regions*								
Any region		60.0	(43.6–74.5)	49.4	(39–59.8)	56.5	(42.3–69.8)	.53
Upper quarter		28.6	(16.3–45.1)	30.6	(21.8–41.1)	34.8	(22.7–49.2)	.82
Lower extremity		25.7	(14.2–42.1)	30.6	(21.8–41.1)	15.2	(7.6–28.2)	.16

Upper quarter: neck, shoulder and thoracic region. Lower extremity: hip, knee, lower leg, foot. *CI* Confidence Interval. A maximum of *n* = 2 missing data in one single body region

### Demographics, health- and work-related outcomes in AF and DS

The self-reported demographics, health- and work-related data for the AF and DS are presented in Table 3. AF were significantly older (*p* < 0.01), shorter (*p* = 0.04), weighed less (*p* = 0.03) and less physically active (p < 0.01) than DS. Tobacco use was more common among the DS (p < 0.01). The AF rated that they were physically prepared to a higher degree than DS (*p* = 0.02).

**Table 3:** Demographics, physical activity level, health- and work-related data of combined air force personnel and deployed soldiers

		Air force		Deployed soldiers		*p*-value
		*n* = 166		*n* = 185		
Age, years	Mean (95%CI)	**39**	(38–41)	**34**	(35–35)	<.01
Height, m		**1.81**	(1.80–1.82)	**1.82**	(1.81–1.83)	.04
Weight, kg		**82**	(81–83)	**84**	(82–85)	.03
BMI, kg/m^2^		25.1	(24.7–25.4)	25.2	(24.9–25.5)	.46
Physical activity level	% (95%CI)					<.01
Low/inactive		**4.3**	(2.1–8.6)	**3.2**	(1.5–6.9)	
Active		**30.5**	(24.0–37.9)	**14.1**	(9.8–19.8)	
Highly active		**65.2**	(57.7–72.1)	**82.7**	(76.6–87.5)	
Physical health	"					
Poor		1.2	(0.3–4.3)	1.1	(0.3–3.9)	.49
Good		56.6	(49.0–63.9)	50.8	(43.7–57.9)	
Excellent		42.2	(34.9–79.8)	48.1	(41.0–55.3)	
Mental health	"					
Poor		0	(0–2.3)	0.5	(0.1–3.0)	.06
Good		28.5	(22.1–35.8)	19.5	(14.4–25.8)	
Excellent		71.5	(64.2–77.9)	80.0	(73.7–85.1)	
Physical environment	"					
Poor		1.2	(0.3–4.3)	1.1	(0.3–3.9)	.27
Good		50.9	(43.4–58.4)	42.7	(35.8–49.9)	
Excellent		47.9	(40.4–55.5)	56.2	(49.0–63.2)	
Social environment	"					
Poor		0	(0–2.3)	1.6	(0.6–4.7)	.06
Good		41.2	(34.0–48.9)	32.4	(26.1–39.5)	
Excellent		58.8	(51.2–66.0)	65.9	(58.9–72.4)	
Work ability	"					
Poor		0.6	(0.1–3.4)	0.5	(0.1–3.0)	.69
Good		25.5	(19.4–32.6)	21.1	(15.8–27.5)	
Excellent		73.9	(66.8–80.0)	78.4	(71.9–83.7)	
Uses smokeless tobacco	"	**21.3**	(15.8–28.2)	**38.8**	(32.0–46.0)	<.01
Smoker-yes	"	**.6**	(0.1–3.4)	**7.8**	(4.7–12.7)	<.01
Motivated-yes	"	100	(97.7–100)	97.8	(94.6–99.2)	.13
Mentally prepared-yes	"	100	(97.7–100)	98.9	(96.2–99.7)	.50
Physically prepared-yes	"	**95.7**	(91.4–97.9)	**88.6**	(83.3–92.5)	.02
Sick-listed -yes	"	10.1	(6.0–16.5)	4.6	(2.1–9.7)	.10

Numbers in bold indicate significant difference between groups (p < 0.05). *BMI* Body mass index, *CI* Confidence Interval

### Demographics, health- and work-related outcomes in FP, HP and RC

Table 4 shows the self-reported demographic, physical activity level, health- and work-related data for the 166 included AF personnel. HP were significantly older than FP and RC. Most regarded themselves as physically active or highly active and rated good to excellent health. They report that they were motivated, as well as mentally and physically prepared for their tasks. Regarding tobacco use, 19 to 23% used smokeless tobacco while only 2% of RC were smokers (none FP or HP).

**Table 4:** Demographics, physical activity level, health- and work-related data of Air force personnel (*n* = 166)

	Mean (95%CI)	Fighter pilots		Helicopter pilots	Rear crew			p-value
		*n* = 35		*n* = 85		*n* = 46		
Age, years		**35**^a^	[33–38]	**42**^a,b^	(40-43)	**38**^b^	[26–40]	<.01
Height, m		1.82	(1.80–1.84)	1.80	(1.79–1.82)	1.80	(1.78–1.81)	.18
Weight, kg		81	(79–84)	83	(81–84)	82	(81–83)	.27
BMI, kg/m^2^		25	[24, 25]	25	[24]	25	[25, 26]	.22
	% (95%CI)							
Physical activity level								.56
Low/inactive		5.7	(1.6–18.6)	3.6	(1.2–10.1)	2.3	(0.4–15)	
Active		31.4	(18.6–48.0)	34.9	(25.6–45.7)	22.7	(12.8–36.0)	
Highly active		62.9	(46.3–76.8)	61.4	(50.7–71.2)	75.0	(60.6–85.4)	
Physical health	"							.16
Poor		5.7	(1.6–18.6)	0	(0–4.3)	0	(0–7.7)	
Good		54.3	(38.2–69.5)	54.1	(43.6–64.3)	63.0	(48.6–75.5)	
Excellent		40.0	(25.6–56.4)	45.9	(35.7–56.4)	37.0	(24.5–51.4)	
Mental health	"							.23
Poor		0	(0–9.9)	0	(0–4.4)	0	(0–7.7)	
Good		40.0	(25.5–56.4)	26.2	(18.0–36.5)	23.9	(13.9–37.9)	
Excellent		60.0	(43.6–74.5)	73.8	(63.5–82.0)	76.1	(62.1–86.1)	
Physical environment	"							.48
Poor		0	(0–9.9)	1.2	(0.2–6.4)	2.2	(0.4–11.3)	
Good		40.0	(25.5–56.4)	53.6	(43.0–63.8)	54.3	(40.2–67.9)	
Excellent		60.0	(43.6–74.5)	45.2	(35.0–55.9)	43.5	(30.2–57.8)	
Social environment	"							.14
Poor		0	(0–9.9)	0	(0–4.4)	0	(0–7.7)	
Good		54.3	(38.2–69.5)	40.5	(30.6–51.2)	32.6	(20.9–47.0)	
Excellent		45.7	(30.5–61.8)	59.5	(48.8–69.4)	67.4	(53.0–79.1)	
Work ability	"							.72
Poor		0	(0–9.9)	0	(0–4.4)	2	(0.4–11.3)	
Good		22.9	(12.1–39.0)	27.4	(19.0–37.8)	23.9	(13.9–37.9)	
Excellent		77.1	(61.0–87.9)	72.6	(62.3–81.0)	73.9	(59.7–84.4)	
Uses smokeless tobacco	"	22.9	(12.1–39.0)	21.4	(14.0–31.4)	20.0	(10.9–33.8)	.97
Smoker-yes	"	0	(0–9.9)	0	(0–4.4)	2.2	(0.4–11.3)	.49
Motivated-yes	"	100	(90.1–100)	100	(95.5–100)	100	(92.0–100)	
Mentally prepared-yes	"	100	(90.1–100)	100	(95.5–100)	100	(92.0–100)	
Physically prepared-yes	"	97.0	(84.7–99.5)	96.4	(90.0–98.8)	93.3	(82.1–97.7)	.60
Sick-listed -yes	"	**16.7**^**c**^	(7.3–33.6)	12.9	(6.9–23.5)	**0**^**c**^	(0–9.4)	.02
	Median (IQR)							
Total air time (hrs)		1300*^‡^	(700–2000)	**2500**^a,b^	(1963–3210)	**900**^b,c^	(240–1587)	<.01
Annual air time (hrs)	"	120	(100–140)	**130**^†^	(95–193)	**100**^†^	(60–120)	<.01

^a^ = significant difference between fighter pilots and helicopter pilots; ^b^ = significant difference between helicopter pilots and rear crew, and ^c^ = significant difference between fighter pilots and rear crew. Sick-listed: Fighter pilots (*n* = 30), helicopter pilots (*n* = 62), Rear crew (*n* = 37). Numbers in bold indicate significant difference between groups. *BMI* Body mass index, *CI* Confidence Interval

### Factors associated with MSD

Table 5 shows the results from the univariate logistic regression analyses. One work-related, two individual and six health-related variables were univariately associated (*p* < 0.20) with upper quarter MSD during the previous 12 months. However, the variable “motivated” was not carried forward to the multiple analysis due to many missing answers (*n* = 5). For the lumbar region, one work-related, two individual and one health-related variable were associated with MSD for the same period.

**Table 5:** Univariate analysis, unadjusted odds ratios (OR) for MSD in the upper quarter of the body and lumbar regions

MSD cases (yes/no)	Upper quarter			p-value	Lumbar region		p-value
	139/211^a^				104/247		
	n	OR	95% CI		OR	95% CI	
Occupation							
Air force personnel	166	**3.44**	2.19–5.39	<.01	**2.15**	1.35–3.43	<.01
Deployed soldiers	185	1.0	(ref)				
Age (years) (cont.)	351	**1.04**	1.01–1.07	<.01	**1.05**	1.02–1.08	<.01
Height (cm) (cont.)	344	**0.97**	0.93–1.00	.07	**1.03**	0.99–1.08	.14
BMI (kg/m^2^) (cont.)	344	0.76	0.92–1.13	.45	0.97	0.87–1.08	.59
Physical activity							
Low/inactive	13	0.91	0.27–3.04	.87	2.10	0.64–6.97	0.22
Active	76	1.0	(ref)		1.0	(ref)	
Highly active	259	0.93	0.51–1.56	.78	1.00	0.57–1.75	.99
Uses Smokeless tobacco							
Yes	106	**0.67**	0.41–1.10	.10	0.71	0.55–1.50	.71
No	240	1.0	(ref)		1.0	(ref)	
Smoker							
Yes	15	0.99	0.34–2.85	.99	1.18	0.39–3.54	.77
No	328	1.0	(ref)		1.0	(ref)	
Motivated							
Yes	341	1.0	(ref)		1.0	(ref)	
No	4	**4.75**	0.49–46.15	. 18	2.46	0.34–17.67	.37
Mentally prepared							
Yes	347	1.0	(ref)		1.0	(ref)	
No	2	Err.			Err.		.99
Physically prepared							
Yes	318	1.0	(ref)		1.0	(ref)	
No	28	1.17	0.54–2.56	.69	.972	0.41–2.29	.95
Physical health							
Excellent	159	1.0	(ref)		1.0	(ref)	
< Excellent	191	**1.90**	1.23–2.95	<.01	**2.11**	**1.31–3.41**	<.01
Mental health							
Excellent	265	1.0	(ref)		1.0	(ref)	
< Excellent	84	**1.52**	0.93–2.50	.10	1.37	0.81–2.31	.24
Physical environment							
Excellent	183	1.0	(ref)		1.0	(ref)	
< Excellent	166	**1.86**	1.21–2.87	.01	1.31	0.82–2.07	.26
Social environment							
Excellent	218	1.0	(ref)		1.0	(ref)	
< Excellent	131	**1.56**	1.01–2.43	.05	1.30	0.81–2.07	.28
Work ability							
Excellent	266	1.0	(ref)		1.0	(ref)	
< Excellent	83	1.29	0.79–2.13	.31	1.21	0.71–2.06	.48

Numbers in bold indicate independent variables that are significantly associated with the dependent variable (p < 0.20), and carried forward to the following multiple analysis. “Motivated” was not carried forward to the multiple analysis due to missing values (n = 5). Upper quarter: neck, shoulder and thoracic region. MSD cases: Reported musculoskeletal disorder in the last year. *BMI* Body mass index, *CI* Confidence Interval. ^a^: Data missing from one soldier regarding shoulder MSD

Table 6 shows the results of the multiple logistic regression analyses. Two variables, employed as AF (OR 3.22 (95% CI, 2.03–5.11), *p* < 0.01) and rating one’s “physical health” as less than excellent (OR 1.94 (95% CI,1.22–3.09) p < 0.01) remained associated with upper quarter MSD in the final model. For the lumbar region, being employed as AF (OR 2.07 (95% CI, 1.24–3.44) p < 0.01), greater age (OR 1.03 (95%CI, 1.00–1.06) *p* = 0.04), taller body height (OR 1.05 (95% CI, 1.00–1.09) p = 0.04), and rating one’s “physical health” as less than excellent (OR 1.94 (95% CI, 1.17–3.20) *p* = 0.01) were all associated in the final model with lumbar region MSD.

**Table 6:** Multiple analysis; initial and final odds ratios (OR) for upper body and lumbar regions MSD

	Upper body region						Lumbar region		
	Initial model		p-value	Final model		p-value	Initial and final model^a^		p-value
	OR	95% CI		OR	95% CI		OR	95% CI	
Occupation									
Air Force personnel	**2.84**	**1.74–4.64**	<**.01**	**3.22**	**2.03–5.11**	**<.01**	**2.07**	**1.24–3.44**	**<.01**
Deployed soldiers	1.0	(ref)		1.0	(ref)		1.0	(ref)	
Age (years) (cont.)	1.02	0.99–1.04	.31				**1.03**	**1.00–1.06**	**.04**
Height (cm) (cont.)	0.98	0.94–1.02	.28				**1.05**	**1.00–1.09**	**.04**
Uses Smokeless tobacco									
Yes	1.14	0.68–1.94	.62						
No	1.0	(ref)							
Physical health									
Excellent	1.0	(ref)		1.0	(ref)		1.0	(ref)	
< Excellent	**1.66**	**1.00–2.76**	**<.01**	**1.94**	**1.22–3.09**	**<.01**	**1.94**	**1.17–3.20**	**.01**
Mental health									
Excellent	1.0	(ref)							
< Excellent	0.84	0.44–1.62	.60						
Physical environment									
Excellent	1.0	(ref)							
< Excellent	1.46	0.87–2.43	.15						
Social environment									
Excellent	1.0	(ref)							
< Excellent	1.31	0.74–2.32	.35						

Numbers in bold indicate independent variables that are significantly associated with the dependent variable (p < 0.05).^a^The initial model remained significant for MSD in the lumbar region. Upper quarter: neck, shoulder and thoracic region; *CI* Confidence Interval

## Discussion

This is the first study to present prevalence of MSD in both the Swedish AF and Swedish army DS and to describe differences and similarities between cohorts. Despite a generally healthy lifestyle, approximately 80% of AF reported occasional or more persistent complaints/injuries in their bodies during the previous year. The most commonly affected region was the upper quarter of the body, with a prevalence of approximately 60%. Thus, our findings are in accordance with previous reports of high rates of neck pain among FP [2, 31, 32] and HP [4, 18, 33], as well as among RC [4, 23]. In line with a meta-analysis including FP, HP and transportation pilots, where no differences in prevalence of neck pain, back pain or degenerative findings were found [19], our AF cohort showed no statistically significant differences in prevalence and distribution of MSD between fighter pilots, helicopter pilots and rear crew.

Over 70% of DS reported MSD in the last year, with the lower extremities accounting for 44%. This supports previous studies which included Swedish army conscripts [16] and marines (51%) [6], where the lower extremities were found to be the most common location for MSD. This is indeed a well-known problem in many other countries, where it has been shown that the lower limbs of British infantry soldiers are commonly affected by MSD [34]. With high loads on the lower extremities in service, overload and acute injuries naturally affect this region in army soldiers. Running and performing sports seem to be common mechanisms for both acute and overload injuries, but combat training is also a common activity performed while being injured [34, 35]. Regarding the lumbar region, the one-year prevalence among our DS cohort was 22% and is comparable to Danish deployed soldiers (26%) [27], but lower compared to Swedish marines (36.0%) [6]. The high rates of back pain among Swedish marines might be due to high loads during combat training [6]. Further, regarding the upper quarter of the body (26%), prevalence among our cohort was slightly lower than that (33%) in the Swedish marines [6].

Regarding comparisons between the DS and AF, the prevalence of lower extremity MSD was surprisingly not significantly higher for DS (44%) compared to AF (35%) in our study. The lack of difference between groups may be partly explained by the somewhat higher than expected prevalence among our AF cohort when compared to that reported for Austrian helicopter pilots and crew [4]. Another important reason for the lack of difference may be the implementation of the MSP for the Swedish army. Aside from the lower extremities, the one-year and point prevalence of MSD, respectively, were significantly higher among AF compared to DS in the upper quarter and lumbar regions. Further, as well as a higher prevalence, self-rated pain intensity was significantly higher for both the upper quarter and lumbar regions among our AF cohort. The prevalence difference may be partly be explained by their older age [3]. The older age was, however, no coincidence since it takes several years of training in the handling of the aircraft and associated systems before they complete their pilot education. Further, HP were significantly older than RC and FP, and had significantly more flight hours logged during their career compared to FP, who had significantly more flight hours logged compared to RC. This difference in age and flight hours were, however, not associated with higher prevalence of MSD within the AF cohort. Different external factors to explain the reported upper quarter MSD in air force cohorts have been suggested. One such factor for neck pain among fighter pilots is the G-forces experienced during flying [19]. Another risk factor is the use of helmet-mounted equipment among helicopter pilots [18, 20] and fighter pilots [22, 36]. The use of helmet-mounted equipment increases the load on the neck and has been linked to increased strain on neck muscles in static laboratory situations [37] and in controlled centrifuge measures [21]. Further, the exposure to disadvantageous postures or work tasks in helicopters has been associated with neck pain in Dutch helicopter pilots and rear crew [23]. Movements, such as neck rotation during G-manoeuvres, were associated with flight-related neck pain in Norwegian fighter pilots [24]. These suggested factors likely contribute to the almost double one-year and point prevalence of upper quarter MSD in AF (54.8 and 31.3%) compared to DS (26.1 and 13.6%) in our study. For the lumbar region, AF (38.0 and 18.7%) reported significantly higher values than DS (22.2 and 7.1%) for both one-year and point prevalence. The prevalence in AF is, however, lower compared to Finnish fighter pilots (71%) [26] and US helicopter pilots and rear crew (77.8%) [3]. One further reason behind the lower prevalence of MSD in DS compared to AF could be that the MSP has already been implemented for the Swedish army. The lower prevalence of MSD might be an effect of the preventive program.

In the logistic regression analyses we included demographic, health- and work-related factors from the MSP questionnaire previously used in studies on Swedish army soldiers and conscripts [14, 16] as potential independent factors related to MSD. The reason for the selection of these possible factors was that previous MSD, physical inactivity, smoking and self-reported lower ratings of mental health have been shown to be important risk factors for premature discharge from service for Swedish conscripts [16]. Our results revealed that being part of the AF (OR 3.22) and rating the state of their physical health to be less than excellent (OR 1.94) were significantly associated with having upper quarter MSD. Being part of the AF (OR 2.07), older age (OR 1.03), taller body height (OR 1.05), and rating the state of their physical health to be less than excellent (1.94) were significantly associated with MSD in the lumbar region. The interpretation of age and body height is that the odds of having MSD increases with 3% for each year and with 5% for each centimetre, respectively. The MSP questionnaire is used as a screening tool to identify individuals with ongoing or past MSD and low health status. The questionnaire may therefore be sub-optimal in determining risk factors in regression analyses. One must always consider the potential for residual confounders in regression analyses [38]. However, we wanted to use the already implemented questionnaire in this study as it is used in daily practice. None of the associated independent factors were considered to confound the results of the logistic regressions in this study.

In line with an earlier study [16], questions about self-rated health, previous and present MSD and work-related factors are important to include in a screening process and should therefore also be included for air force personnel. Also, it is important to notice that being part of the AF was associated with higher prevalence of upper quarter MSD. Together, our findings and those from earlier studies stress the importance of a systematic screening program for air force personnel. The reduced discharge rate from basic military training in Swedish conscripts by screening recruits using a questionnaire and physical tests [14] indicates benefits of yearly musculoskeletal screening also among Swedish AF. We suggest that screening of air force personnel using the MSP should include questions about general health and prevalence of MSD in specific body regions.

The physical load on the spinal structures of pilots has increased due to an increase in the length of missions, greater levels of G-forces and development of head-worn equipment. Earlier interventions for pilots have included exercise programs that aim to improve flexibility, strength and/or sensorimotor control of the neck muscles [32, 39, 40]. The fact that there is still a high prevalence of MSD in the upper quarter of the body, and that the MSP has been found to be effective in reducing prevalence of MSD in the lower extremities in the army, further strengthens the importance of developing a protocol also for the AF. Importantly, however, since the upper quarter and lumbar regions were the most commonly reported body regions for experiencing pain among the AF, the MSP has to be adapted to AF with other tests for these regions. We suggest that for the AF, tests of muscular flexibility, strength, endurance and of sensorimotor control targeting the upper quarter as well as the lumbar regions should be used in future studies. These studies should establish whether functional limitations in these regions can be detected and which tests, if any, should be added to a protocol aiming to reduce the high numbers of MSD in Swedish AF.

### Methodological considerations

We have a few methodological considerations. Firstly, there is a need to discuss the generalizability of the AF group to other air force personnel and the DS group to army soldiers. The AF group can be considered fully representative since the data collection included almost all male employed air force personnel at two Swedish air bases and included fighter pilots, helicopter pilots and rear crew. From the first air base, all HP and RC in duty were included during 2013. From the second air base, all HP and RC together with FP were included during 2016. No major significant differences were found between the two data collection periods regarding MSD, health- and work-related factors. However, the reported prevalence of MSD for the neck, but not the combined upper quarter of the body, was higher and the annual flight time was lower for the latter data collection. The DS were employed as army soldiers or officers who were undergoing medical checks in preparation for deployment. They had not been pre-selected to deployment and therefore they were not considered healthier than other Swedish army soldiers or had less injuries. However, data collection was performed during a time when they were preparing for deployment, the setting in which they answered their questionnaires could potentially have influence their answers. Further, in the statistical analyses, we included only participants that were older than 25 years of age. By including an age-relevant sample from the DS, this group may not be completely representative of DS in general. However, this was necessary in order to compare this group to the AF. The reason for excluding DS that were younger than 25 years of age was that we aimed for similar age in the analyses since age is a factor known to be associated with MSD [3].

Secondly, the use of questionnaires rather than medical records is sometimes questioned. However, when using medical records, there is always a risk of underestimating true prevalence. We do not believe the numbers are excessive, since underreporting of musculoskeletal injuries among US Army soldiers has been reported [41]. Therefore, in line with previous research, musculoskeletal pain [4, 18, 33] or musculoskeletal complaints or injuries [14] (i.e. MSD) are reported in the questionnaire. Thirdly, several of the variables were collapsed into fewer categories. Regarding MSD, we combined the neck, the thoracic region and the shoulders into “upper quarter of the body” in our analyses because several muscles have their origin in the neck and insert in the shoulder or thoracic region areas and high proportions of co-morbidity in these regions exists. For general health, the seven-point scale was collapsed and coded into: poor (≤3), good [4, 5], or excellent (≥6) for descriptive analyses. For logistic analyses, the answers were dichotomized into: less than excellent and excellent. The reason for this was that it should be easier to compare data with earlier reports [14] using the same questions. and that a less than excellent rating can be considered a risk [29]. Fourthly, we chose to use MSD during the previous year in the upper quarter and lumbar regions as the main outcome variables for the logistic regression analyses. With the cross-sectional design in mind, there can be a risk to underestimate the true prevalence of MSD if “MSD at present” (i.e. point prevalence) were used, since pain episodes often fluctuate [13]. No association between work or leisure time and MSD were made, and we therefore do not know whether their MSD were caused by work or other factors. This is, however, not necessarily valuable information, since personnel answering positively to these questions are further assessed by the units/air bases´ physiotherapists. Lastly, our findings should be interpreted with the cross-sectional design in mind, i.e. no conclusions on causal effects can be drawn. This design, however, was applicable to answer the research question.

## Conclusion

Despite a generally healthy lifestyle, most of the Swedish AF and army DS reported MSD. Considering that the implementation of the MSP for Swedish air force personnel is absent but is under development, the results of this study indicate that the MSP can be a meaningful tool to prevent MSD in air force personnel.

## Data Availability

There are ethical restrictions regarding data availability for public release in this study since identification of participants from the data cannot be ruled out. Data contained in this paper are considered as sensitive. According to the Ethical committee in Sweden, and within the Swedish Armed Forces, we are not allowed to have data available for public release due to ethical restrictions. We can only make the data available upon reasonable request, which will also involve discussions with the Swedish Armed Forces. Contact information: Swedish Armed Forces. Research coordinator. Anders Claréus. 107 85 Stockholm/Sweden. Phone: + 46 8 788 85 26. E-mail: anders.clareus@mil.se

## Abbreviations

AF: Air force personnel
ANOVA: Analysis of variance
BMI: Body mass index
CI: Confidence intervals
DS: Deployed soldiers
FP: Fighter pilots
HP: Helicopter pilots
IQR: Interquartile range
MSCI: Musculoskeletal complaints and injuries
MSD: Musculoskeletal disorders
MSP: Musculoskeletal screening protocol
NPRS: Numerical pain rating scale
OR: Odds ratio
RC: Rear crew
SD: Standard deviation
