# Correction to: Musculoskeletal disorders and their associations with health- and work-related factors: a cross-sectional comparison between Swedish air force personnel and army soldiers

**DOI:** 10.1186/s12891-020-03677-5

**Published:** 2020-10-10

**Authors:** Matthias Tegern, Ulrika Aasa, Björn O. Äng, Helena Larsson

**Affiliations:** 1 Department of Community Medicine and Rehabilitation, Unit of Physiotherapy, Umeå University, Umeå, Sweden; 2 Department of Neurobiology, Care Sciences and Society, Division of Physiotherapy, Karolinska Institutet, Huddinge, Sweden; 3 School of Education, Health and Social Studies, Dalarna University, Falun, Sweden; 4 Centre for Clinical Research Dalarna, Uppsala University, Falun, Sweden; 5 Swedish Armed Forces, HQ, Stockholm, Sweden

**Correction to: BMC Musculoskelet Disord 21, 303 (2020)**

**https://doi.org/10.1186/s12891-020-03251-z**

Following publication of the original article [1], the authors noticed that the numbers 1–5 in the Methods section under subsection “Health- and work-related questions” were mistakenly interpreted as references.

Health- and work-related questions

“To assess their self-rated general health, the following questions were used: How do you experience your [1] “physical health” [2] “mental health” [3] “social environment” [4] “physical environment” and [5] “work ability”? A seven-point scale with answers ranging from “very poor” to “excellent, cannot be better” was used. In the analyses, the answers were collapsed and coded into: poor (≤3), good [4, 5], or excellent (≥6) according to Larsson et al. [16].”

The original article [1] has been updated.
